# Clinical development of imatinib: an anticancer drug

**DOI:** 10.4155/fso.15.92

**Published:** 2016-01-25

**Authors:** Dipanjan Goswami, Sanjay Gurule, Abhiroop Lahiry, Amit Anand, Arshad Khuroo, Tausif Monif

**Affiliations:** 1 Department of Clinical Pharmacology & Pharmacokinetics, Sun Pharmaceutical Industries Ltd, HSIDC, GP-5, Old Delhi Gurgaon Road, Udyog Vihar Industrial Area, Gurgaon 122 015, Haryana, India

**Keywords:** bioavailability study, imatinib mesylate, method development, multiple reaction monitoring ISR, validation

## Abstract

**Background:**

A novel and accurate high-throughput tandem mass spectroscopic method has been developed and validated for determination of imatinib, a *protein-tyrosine kinase* inhibitor against chronic myeloid leukemia.

**Materials & methods:**

Chromatographic separation was carried on XTerra^®^ RP18 column (150 mm × 4.6 mm, 5 µm particle size) manufactured by Waters Corporation, MA, USA. The detection was performed on a triple quadruple tandem mass spectrometer by multiple reactions monitoring mode via electrospray ionization source.

**Results:**

The selective and sensitive method was linear in the concentration range of 9.57–4513.29 ng/ml and reported no matrix effect.

**Conclusion:**

The mean C_max_ was found to be 10–15% lower in European subjects as compared with Indian subjects.

## Abstract (lay abstract)

**Lay abstract:** We describe a new approach to determine the concentration of imatinib in plasma samples obtained after the administration of a reference and a test product of a 400-mg dose. The study was conducted to explore racial effects, involving healthy volunteers from India and Europe. The study was done after an approval of protocol and informed consent form Jamia Hamdard Institutional Ethics Committee (JHIEC), New Delhi, India. This study has been done for the first time on the two different races. The findings highlight that the bioavailability in Indian volunteers was comparable to that of European volunteers.

**Figure 1.**
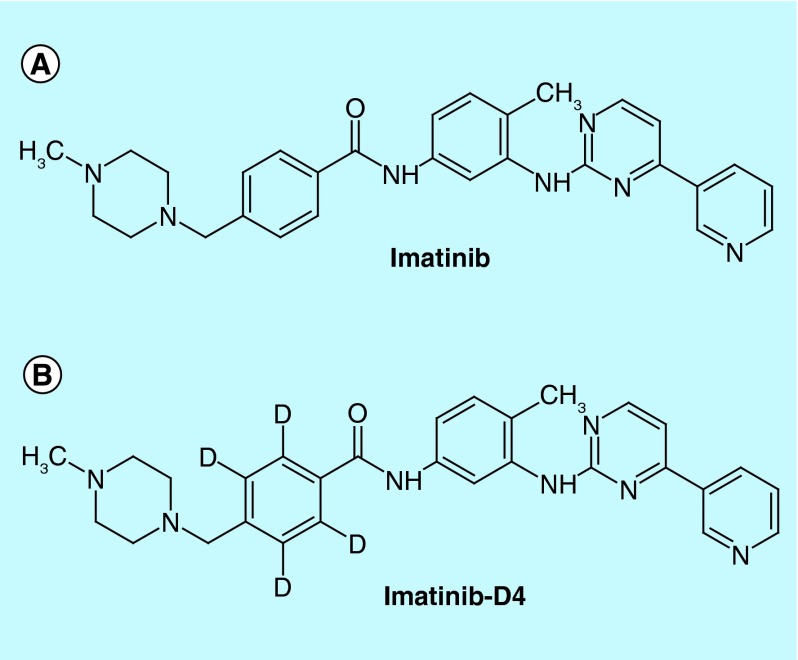
**Chemical structures of analyte and internal standard.** Chemical structures of **(A)** imatinib **(B)** imatinib-D4.

**Figure 2.**
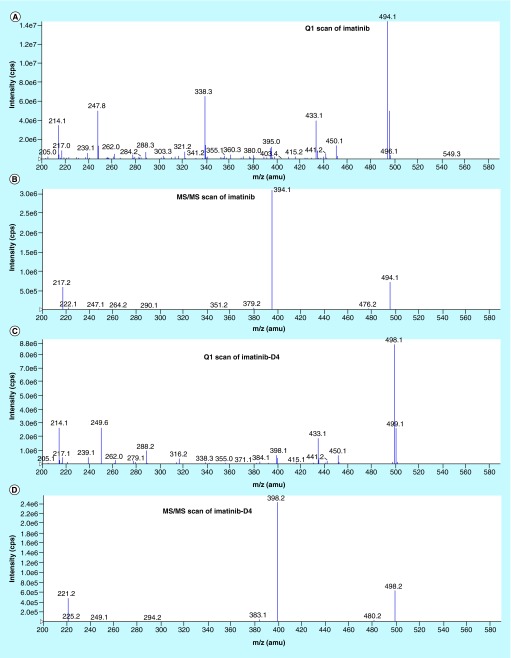
**Tandem mass spectrometric scan of analyte and internal standard.** **(A)** Parent ion (Q1) scan of imatinib; **(B)** product ion (MS/MS) scan of imatinib; **(C)** parent ion (Q1) scan of imatinib-D4; **(D)** iroduct ion (MS/MS) scan of imatinib-D4. amu: Atomic mass unit; cps: Counts per second; MS/MS: Tandem mass spectrometry.

**Figure 3.**
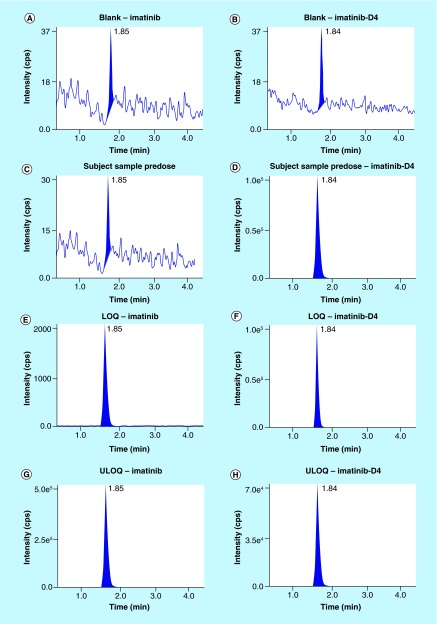
**Chromatograms.** **(A)** Chromatogram of blank at retention time of imatinib, **(B)** chromatogram of blank at retention time of imatinib-D4, **(C)** chromatogram of subject sample predose, **(D)** chromatogram of subject sample predose-imatinib-D4, **(E)** chromatogram of LOQ – imatinib, **(F)** chromatogram of LOQ – imatinib-D4, **(G)** chromatogram of ULOQ – imatinib, **(H)** chromatogram of ULOQ – imatinib-D4. LQC:Low quality control; ULOQC: Upper limit of quantification.

**Figure 4.**
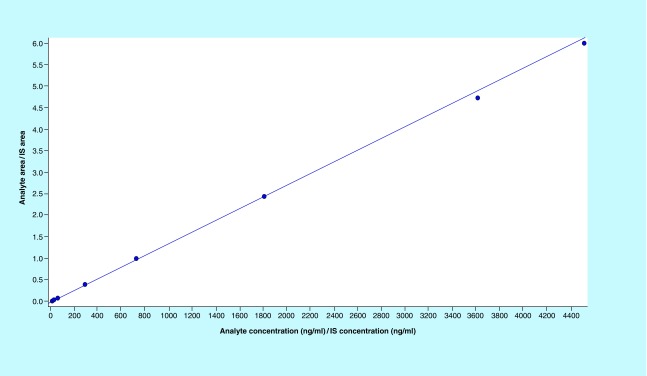
**Calibration curve of imatinib.** Method was found to be linear in the range 9.57–4513.29 ng/ml. IS: Internal standard.

**Figure 5.**
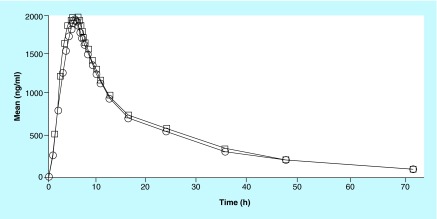
**Pharmacokinetic profile of imatinib (test and reference) in Indian volunteers.** Circle: Reference; Rectangle: Test.

Imatinib mesylate, (4-[(4–Methyl–1–piperazinyl) methyl]-N-[4–methyl–3-[(4-[3–pyridinyl]-2–pyrimidinyl) amino]-phenyl]benzamide methanesulfonate), a tyrosine-kinase inhibitor, is used to treat chronic myelogenous leukemia (CML), gastrointestinal stromal tumors (GISTs) and a number of other malignancies [1–6]. Imatinib mesylate is a protein-tyrosine kinase inhibitor that inhibits the Bcr-Abl tyrosine kinase, the constitutive abnormal tyrosine kinase created by the Philadelphia chromosome abnormality in chronic myeloid leukemia (CML). Protein-tyrosine kinases play a fundamental role in signal transduction, and deregulated activity of these enzymes which has been observed in cancer and benign proliferative disorders [7]. Clinical studies from Phase I and Phase II trials with imatinib in patients, suffering from chronic myeloid leukemia (CML) with interferon (IFN) resistance or from advanced Philadelphia chromosome-positive leukemias showed antitumor activity. This activity is based on overall hematologic and cytogenetic response rates with this single-agent treatment of imatinib at well tolerated doses. Imatinib also inhibits the receptor tyrosine kinases for PDGF and SCF – called c-kit [8–13].

The pharmacokinetic data for imatinib are interesting. Imatinib is almost completely absorbed (98%) after oral administration, which indicates high solubility, high permeability and low hepatic extraction ratio. T_max_ is achieved in 2–4 h. Food has no relevant impact on the rate or extent of bioavailability of imatinib. The absorption of imatinib is marginally affected by administration with fat-rich food, with an 11% reduction in C_max_, a 1.5-h prolongation of T_max_ and a 7.4% reduction in AUC. The use of antacids has not been found to significantly affect imatinib absorption [14].

There are significant data available on analytical method for determination imatinib [15–20].

A reliable, selective and high-throughput method, for plasma estimation of imatinib using imatinib-D4, as an internal standard (IS), therefore, has been presented in this paper. The dynamic linearity ranges, high extraction recovery, stability in blood and plasma samples have been discussed. One of the advantages of this high-throughput bioanlytical method was achieving recovery greater than 85%. Absolute MF and IS normalized MF was evaluated. The results of Matrix Factor showed that ion suppression or enhancement from the plasma matrix components was nullified for the first time to the best of the knowledge of the authors of this paper. Exhaustive stability studies have been carried out, considering the separation of imatinib inactive metabolites, N-desmethyl imatinib and N-Oxide. The method development was challenging as N-desmethyl imatinib and N-oxide of imatinib were interfering with the parent compound. Therefore, method was developed in a way to separate N-desmethyl imatinib and N-oxide of imatinib from parent compound and prevent its back conversion to parent compound. As regulatory agencies (US FDA and EMEA guideline) state that only parent compound should be analyzed and it is not mandatory to analyze N-desmethyl imatinib and N-oxide imatinib.

The selection of the lowest limit of quantification has been based on elimination profile capturing at least five half-lives. The validated method was used for exploratory bioavailability study of a single dose of GLEEVEC (imatinib mesylate) 400 mg tablets (containing 400 mg of imatinib [as imatinib mesylate]) of Novartis Pharmaceuticals Canada Inc., Canada. The Test formulation (containing 400 mg of imatinib [as imatinib mesylate]) of Sun Pharmaceuticals was also administered to Indian Subjects with special note on exploration of racial effects, involving the healthy volunteers from Europe as well.

## Experimental

### Chemicals & apparatus

Working standards of imatinib (98.07% purity) and its internal standard imatinib-D_4_ (deuterium labeled imatinib; 99.67% purity) were procured from Vivan Life Sciences India (Figure 1). Ammonium formate, methanol and orthophosphoric acid were purchased from Qualigens Fine Chemicals (a division of GSK Ltd, Mumbai India). Water was purified using a Milli-Q device (Millipore, Mosheim Cedex, France). Different individual lots of human plasma containing tripotassium ethylene diamine tetra acetic acid (K_3_EDTA) were collected from Yash Laboratories (Delhi, India). Glass shell vials (1.2 ml) were procured from Chromatopak, (Mumbai, India).

### Instrumentation

The liquid chromatographic separation of imatinib was achieved on a HPLC from Shimadzu Scientific Instruments (Shimadzu Corporation, Kyoto, Japan) equipped with two LC–20AD delivery pumps, an online DGU–20A3 prominence solvent degasser, a SIL-HTc Shimadzu autosampler and a CI3M–20A prominence column oven; using XTerra^®^ RP18 analytical column (150 × 4.6 mm, 5 μm) eluted with mobile phase at a flow rate of 1.000 ml/min. An injection volume of 10 µl was used for each analysis. The column oven and sample cooler temperature were maintained at 35°C ± 1°C and 5°C ± 1°C, respectively. All the chromatographic solvents were degassed before use. MDS, Sciex API 3200 mass spectrometer was operated using turbo ion spray, operated in the positive ion mode. Analytes were detected by tandem mass spectrometry using multiple reaction monitoring (MRM) of precursor-product ion transitions with 400 ms dwell time, at *m/z* 494.1/394.1 for imatinib and *m/z* 498.1/398.2 for imatinib-D4.

The compound and source parameters, optimized by infusing a solution of imatinib and imatinib-D4 (˜ concentration of 50 ng/ml each) into the mass spectrometer, were as follows: ion spray source temperature at 550°C, curtain (CUR) gas at 40, nebulizer gas (GS1) at 50, heater gas (GS2) at 50, ionspray voltage (IS) at 5000 eV and collision-activated dissociation (CAD) at 3; declustering potential (DP) at 65 eV, entrance potential (EP) at 6 eV, collision energy (CE) at 38 eV, and collision cell exit potential (CXP) at 5 eV, for both imatinib and imatinib-D4. Ultra high pure nitrogen was used as GS1, GS2, collision-activated dissociation (CAD) and curtain gas. Quadrupole 1 and 3 were maintained at unit mass resolution.

Calibration curves were constructed by calculating the analyte to IS peak area ratio (y) against analyte concentrations (x). Data acquisition and processing were performed using Analyst version 1.4.2 software (Applied Biosystems, Foster City, CA, USA).

Cleanert PEP–3 SPE, 30 mg/1ml, solid phase cartridges were obtained from Agela technologies (Tianjin, China). Centrifuge 5810R (Eppendorf, Hamburg, Germany) used of solid phase extraction. Zymark Turbovap LV concentrator (Caliper Life Sciences, MA, USA) was used for evaporation. Liquid chromatographic separation was performed using Shimadzu scientific instruments (Shimadzu Corporation, Kyoto, Japan) on XTerra RP18 column (150 mm × 4.6 mm i.d., 5 µm particle size; Waters). Samples were analyzed with API–3200 triple quadrupole mass spectrometer (MDS, Sciex®, Foster City, CA, USA).

### Preparation of stock solutions

The primary stock solution of imatinib (1 mg/ml) and imatinib-D4 (1 mg/ml) was prepared by dissolving the standard compound in methanol. Separate stock solutions were used to prepare calibration curve (CC) standards and quality (QC) control samples. The prepared stock solutions were protected from light and stored in refrigerator between 1–10°C until use. A fresh working solution of IS (1500.00 ng/ml) was prepared everyday by taking appropriate dilution of the stock solution in methanol:water (50:50 v/v). CC and QC working solutions for imatinib were prepared by serially diluting the stock solution with methanol:water (50:50 v/v).

### Preparation of plasma CC standards & QC samples

Plasma standards were prepared in human K_3_EDTA plasma by spiking 2% v/v of the working solutions for a concentration range of 9.57–4,513.29 ng/ml for imatinib. Similarly, QCs were prepared at lower limit of quantitation (LOQQC) 9.58 ng/ml; low level (LQC) 23.41 ng/ml; medium level (MQC) 1,800.91 ng/ml and at higher limit of quantitation (HQC) 3,601.82 ng/ml, for imatinib. The spiked CC and QC samples were prepared as a bulk (i.e., bulk spiking) at room temperature and were divided into aliquots in polypropylene tubes. All samples were prepared room temperature and stored in the freezer at below -15°C until analysis.

### Plasma sample preparation

A solid phase extraction (SPE) method was developed to isolate imatinib from plasma. The requisite plasma samples were retrieved from freezer room and thawed at room temperature. Plasma samples were prepared by mixing 50 µl of IS (1,500.00 ng/ml) working solution with 0.100 ml aliquots of human plasma sample in polypropylene tubes. The contents of polypropylene tubes were mixed, after which 400 µl of 1.25% orthophosphoric acid in HPLC Grade water was added to each sample.

Following vortexing, the pretreated samples were loaded using an eppendorf pipette on Cleanert PEP–3 SPE cartridges. Before proceeding for sample loading, SPE cartridges were conditioned with 0.5 ml of methanol followed by 0.5 ml of HPLC-grade water. Postloading phase, the Cleanert PEP–3 SPE cartridge was washed with 1 ml washing solution, that is, methanol – water (5:95, v/v). The analyte and IS was eluted off the cartridges with 1 ml of methanol twice. The extraction was carried out on centrifuge by running for 1 min at 4,000 rpm after each addition. Eluents were evaporated to dryness at 50°C and at about 20 psi under a stream of dry nitrogen using Zymark Turbovap LV concentrator. Then, dried residue was reconstituted with 400 µl of mobile phase and transferred into Chromatopak 1.2 ml glass shell vials. Finally 10 µl of each sample was injected into the column for analysis.

## Method validation

Method validation was conducted according to the US FDA/EMA guidance document on validation of bioanalytical methods [21–23]. Method was validated in terms of lineraity, selectivity, matrix effect, matrix factor, extraction recovery, dilution integrity, intra/interday precision and accuracy and stability.

Selectivity was carried out using eight different lots of K_3_EDTA blank human plasma (six normal, one lipemic and one hemolyzed), processed by same extraction method and analyzed to determine the extent to which endogenous substances may contribute to the interferences for analyte and IS. Linearity of method was assessed by constructing calibration curve with different concentrations ranging from 9.57 to 4,513.29 ng/ml. The linearity of bioanalytical method was determined by plotting area ratio of analyte/IS against theoretical concentration of analyte. The within- and between- (intra and inter) batch precision and accuracy were determined by analyzing six replicates of QC samples produced at four different concentration levels; that is, LOQQC, LQC, MQC and HQC, each in a batch and on three different batches, respectively. The extraction recovery was obtained by comparing the peak area of six replicates of the drug spiked in human plasma produced at three different concentration levels, that is, LQC, MQC and HQC with the working solution of equivalent concentration of LQC, MQC and HQC.

The stability of spiked samples during handling and storage was evaluated in terms of bench top stability, bench top stability during extraction, freeze thaw stability (three cycles of freeze- thaw, freezing was conducted below -15°C and below -50°C and thawing at room temperature), Auto sampler stability of 100.87 h, for long-term stability for 183 days was samples were kept below both -15°C and -50°C. Stock solution stability of both analyte and internal standard was carried out for 21 days at refrigerated temperature (1–10°C). Short-term stability both analyte and internal standard was carried out for 8.46 h at room temperature.

## Application of the method

The validated method was applied to a multicenter bioavailability study. Twenty-six male human volunteers from Delhi and NCR, India and thirty male human volunteers from Terapia, Europe completed the study. Subjects meeting the inclusion and exclusion criteria (Box 1) were enrolled in the study and no waivers were permitted. The Table 1 delineated the demographic profile of volunteers completing the study (Indian and European). Volunteers were admitted at least 11 h before dose administration and were discharged approximately 24 h after administration of the test or reference products during each period of the study. Subjects were asked to make three visits to Clinical Pharmacology Unit (CPU) at 36, 48 and 72 h postdose in each period of the study (Table 2).

The Investigator or his/her designate, informed the subjects before initiation of study through an oral presentation regarding the purpose, procedures to be carried out, potential hazards and subjects rights including the rights to contact Sun Pharma in order to claim compensation in case of trial related injury or death. Subjects were required to understand and sign/initial in the declaration of ICF prior to admission for the study in Period I. The whole informed consent process was documented through audio visual means maintaining the principle of confidentiality.

The protocol and the corresponding informed consent form (ICF) used to obtain informed consent of study subjects were reviewed by the Jamia Hamdard Institutional Ethics Committee (JHIEC) and the studies were initiated only after approval of protocol and the ICF. The committee is constituted and operates in accordance with the Principles and requirements regarding the obligations of investigators and the Ethical Guidelines for Biomedical Research on Human Participants issued by Indian Council of Medical Research (ICMR), New Delhi, and Good Clinical Practices guidelines for Clinical Research in India issued by Central Drugs Standard Control Organization, Ministry of Health and Family Welfare, Schedule Y, Drug & Cosmetics Acts (amended 2005), and ICH (International Conference on Harmonisation) E6 ‘Guidance on Good Clinical Practice’ and the principles enunciated in the Declaration of Helsinki (59th WMA General Assembly, Seoul, October 2008), Therapeutic Products Directorate (TPD) guideline and all other pertinent requirements of 21 CFR (Code of Federal Regulations) Part 320’ of FDA regulatory. All relevant procedures were compiled to ensure that all associates, assisting in the conduct of study, were informed regarding their obligations.

GLEEVEC (imatinib mesylate) 400 mg tablets (containing 400 mg of imatinib [as imatinib mesylate]) of Novartis Pharmaceuticals Canada Inc., Canada, and test formulation (containing 400 mg of imatinib [as imatinib mesylate]) of Sun Pharmaceuticals were administered to Indian volunteers. In addition, an exploratory study was done to find bioavailability of reference product in 30 European subjects at the same dose as that given to Indian subjects. Medication was administered with 240 ml of water under overnight fasting condition. Effect of food was not studied as food has negligible effect on imatinib absorption on healthy volunteers [14] as well as on patients [24], although labeling information recommends taking imatinib with food. Hence, to identify formulation-related effects, if any, while evaluating pharmacokinetic parameters of comparator (i.e., generic drug), imatinib was administered after overnight fast of at least 10 h. Blood samples were collected at predose (duplicate) and at 0.5, 1.0, 1.5, 2.0, 2.5, 3.0, 3.5, 4.0, 4.5, 5.0, 5.5, 6.0, 7.0, 8.0, 9.0, 10.0, 12.0, 16.0, 24.0, 36.0, 48.0 and 72.0 h after drug administration. After each blood sampling, plasma was separated by centrifugation at 4,000 rpm for 15 min and stored below –15°C, until assayed.

The incurred sample reanalysis (ISR) experiment was assessed using a total of 120 samples, selected from single dosed biostudies in fasting conditions. The study samples from a C_max_ time point and elimination phase (at least three times of LLOQ concentration) comprising of 80% sample set and the remaining 20% as random sample time points were selected.

The pharmacokinetics parameters of imatinib was performed using noncompartmental pharmacokinetic method using WinNonlin^®^, Version 5.3 software package (Pharsight Corporation California). The noncompartmental analysis was performed using standard methods for each subject. The area under the plasma concentration-time curve (AUC) was calculated using the trapezoidal rule and extrapolated to infinity.

## Results & discussion

### Method development

To develop a rapid, selective and sensitive method, mass acquisition parameters, chromatographic conditions and an efficient extraction procedure are mandatory. Mass spectrometry parameters can play an important role in selectivity and sensitivity while chromatography has an impact on selectivity and run time of the method. Simple and efficient extraction procedure can help to obtain clearer samples for quantitative recoveries. Selection of internal standard is equally important as it constantly takes care of the analytes for quantification.

#### Mass spectrometry

During the early stage of method development, ESI ionization source offered much higher signal intensities for both analyte and internal standard, infusing the scanning solution (50 ng/ml) for both the analyte and internal standard in mobile phase to acquire optimum mass spectrometeric parameters. Using ESI as ionization source, tuning was performed in positive mode as both the compounds have the ability to accept protons and give protonated species [M+H]^+^ ions in Q1 mode. Hence, the protonated parent ions for imatinib and imatinib-D4, were yielded at *m/z* 494.1 and 498.1 (Figure 2), respectively. The [M+H]^+^ ions from imatinib and imatinib-D4 were selected as the precursor ion and subsequently fragmented in MS/MS mode to obtain the product ion. The fragment ion at *m/z* 394.1 and 398.2 (Figure 2) were produced as the prominent product ion for imatinib and imatinib-D_4_, respectively. The quantification of analytes was performed using the MRM mode due to the high sensitivity of MRM data acquisition, where the precursor and the product ions are monitored. Selecting appropriate dwell time for avoiding cross-talk with imatinib and imatinib-D4 was critical. The cross-talk in MS/MS was observed with 100 ms dwell time while monitoring imatinib and imatinib-D4 in MRM mode. Finally, 400 ms dwell time was found appropriate and no cross-talk could be traced for imatinib and imatinib-D4 between their MRMs and peak shapes also improved significantly. The curtain gas, having 40psig, was found suitable. Efficient mobilization of ions took place at 50 psig for gas 1 (nebulizer gas) and gas 2 (heater gas). The capillary temperature of ion source and ion spray voltage had significantly controlled charge competition between imatinib and imatinib-D4. Therefore, ion source temperature was optimized at 550°C and ion spray voltage set at 5,000eV Table 2.

#### Chromatography

After finalizing mass parameters, it was imperative to optimize liquid-chromatographic conditions to achieve maximum separation between analytes and metabolites. Various combinations of mobile phase and columns were tried to achieve the desired peak-response with acceptable chromatography, maintaining high-throughput analysis output. The diverse combinations of acetonitrile, methanol and buffers (ammonium acetate, ammonium formate and formic acid) were tried to optimize the ratio of mobile phase components, yielding the desired sensitivity and peak shape. The combination of acetonitrile and 10 mM ammonium acetate solution in the ratio of 80:20 showed peak tailing and peak broadening. Methanol revealed the higher peak area response from mass spectrometry, along with improved peak shape, in comparison to acetonitrile, used in mobile phase. Methanol and 10 mM ammonium acetate solution in the ratio of 80:20, displayed slightly better peak shape, however, peak broadening was still observed. Therefore, buffer was changed to ammonium formate. It was found that the combination of methanol and 10 mM ammonium formate solution in the ratio of 85:15, v/vis best suited composition for mobile phase to achieve optimum system response.

#### Column selection

A number of reversed-phase C18 columns, such as Ascentis RP-Amide (150 × 4.6 mm, 5 μm), Phenomenex Synergi Max RP 18 (150 × 4.6 mm, 5 μm), Zorbax SB-C18 and XTerra RP18 were tested to obtain optimal response, suitable retention time and good peak shapes for analyte and IS. Although retention of analytes was found in most of the columns, interference from endogenous compounds could not be eliminated completely. Tailing was observed with Ascentis RP-Amide. The tailing and peak broadening were also noted with Zorbax SB-C18. Results comparable to Ascentis RP-Amide was obtained with Phenomenex Synergi Max RP 18, however, peak tailing was more with Phenomenex Synergi Max RP 18. Desired chromatography of imatinib was achieved with XTerra RP18 analytical column (150 × 4.6 mm, 5 μm) and above-mentioned mobile phase composition. XTerra Columns combine the best properties of silica and polymeric bonded phases with patented Hybrid Particle Technology that replaces one out of every three silanols with a methyl group during particle synthesis. The presence of fewer residual silanols in XTerra columns give exceptionally sharp, high-efficiency peaks for basic compounds.

The flow rate, optimized at 1.000 ml/min, restricted the analysis run time within 5 min.

#### Sample extraction

During method development diverse mechanisms were evaluated to optimize sample extraction. Initially protein precipitation with methanol and acetonitrile was evaluated followed by liquid–liquid extraction (using ethyl acetate, dichloromethane and different combination dichloromethane and isopropyl alcohol). Both protein precipitation and liquid–liquid extraction mechanism yielded low extraction recovery. Additionally, the results showed that there were a cluster of components co-eluting with imatinib suggesting potential of phospholipid-based matrix effects. Since attempts to modify chromatographic conditions to separate analyte from interference component were not successful. Solid phase extraction (SPE), using multiple set of cartridges (HLB, Bond Elut Plexa, PEP–3 SPE and Strata-X) was tried. Improved extraction recovery was observed with Cleanert PEP–3 SPE cartridges only. These cartridges have unique mix mode characteristic (hydrophilic vinyl pyrrolidone and hydrophobic polydivinyl benzene moieties) that provided an excellent capacity to retain analytes of wider polarity spectrum. Moreover, plasma samples was pretreated with 400 µl of 1.25% orthophosphoric acid solution that led to breaking of drug-protein binding and maximized analyte/s retention on hydrophilic hydrophobic balance stationary phases. After loading the cartridges were washed with HPLC Grade water. Different elution solvents such as methanol, acetonitrile and acetone were tried to get rid of unwanted co-eluting matrix components and best results were obtained using acetone as elution solution. Matrix effect is nullified and for the first time by using acetone proved to be best eluting solvent during plasma sample extraction phase for 1, 4–dihydropyridines class of compound. The quantitative expression of matrix effect in terms of matrix factor provided convincing proof of least matrix interference of imatinib and its metabolite components for the first time.

### Method validation

#### Selectivity in presence of other analyte

Area observed in all the lots of blank plasma was less than 20% of the LOQ area, and the area observed at the retention time of IS was less than 5% the area of IS concentration used in the sample preparation as presented in Table 3. Figure 3 shows the typical chromatograms of a blank plasma, blank plasma spiked with IS (Zero sample), a spiked plasma sample with imatinib at Limit of quantification (LOQ) and Upper limit of quantification (ULOQ) level. No interfering peaks from endogenous compounds were observed at the retention times of the analyte and IS. It was also found that there was negligible/null interference from imatinib N-oxide due to use of ESI source. Since N-oxides are known to undergo deoxygenation during APCI mass spectrometry to avoid in-source fragmentation of imtanib N-oxide, ESI source was used. The back conversion of imtaninb–N oxide to imtatinib has been controlled. Moreover, interference from isomeric hydroxylated metabolite has been established using the selective ion pairs for imatinib detection in presence of other analytes. In sum, the chromatograms presented in Figure 3 indicated that the newly developed method is highly selective and free from interferences. The implementation of solid phase extraction method provided a very good selectivity for the analysis of imatinib and IS in the blank human plasma. All the chromatograms were Gaussian in shape and was deemed acceptable.

#### Linearity & range

The calibration curve was found to be linear from 9.57 ng/ml to 4513.29 ng/ml. 1/X^2^-weighted calibration curve is shown in (Figure 4). R^2^ was found to be 0.9997 (Figure 4).

#### Precision & accuracy

Both intra and inter batch CV values ranged from 1.4 to 3.6% for imatinib. The accuracy for imatinib ranged from 97.04 to 102.77%. Table 4 shows a summary of within and between batch precision and accuracy data for QC samples containing imatinib. The recovery found at LQC, MQC and HQC level was 80.86, 93.59 and 87.57% for imatinib, respectively.

#### Recovery

The % mean recovery of all three QC levels was 87.338% whereas the % mean recovery of IS was 87.87% with a %CV of 7.29% and 1.80%, respectively. The CV for all the levels was below 20% as presented in Table 5.

#### Dilution integrity

Dilution integrity samples were meeting the acceptance criteria for accuracy (100±15%) and %CV (<15%). The back-calculated concentrations for dilution controls were in agreement with the theoretical (prepared) concentrations; the accuracy was 99.20% and 100.19% with a %CV of 2.90 and 2.96% after two- and fourfold dilution with blank plasma, respectively.

#### Matrix factor

The suppression or enhancement of analyte and IS response due to matrix components for example lysophosphatidyl choline (*m/z* 496/184) and phosphatidyl choline (*m/z* 758/184), among others. [12]. were assessed by estimating the matrix factor across all QC concentration levels. The mean absolute matrix factor at the low, medium and high QC concentration from six lots of plasma samples was 1.013, 1.027 and 1.007, respectively (Table 6). The CVs of absolute MF and IS normalized MF from six lots of plasma samples were <2%.

#### Stability

The results for all the stability exercises obtained were well within the acceptable limits (%CV ≤15% and accuracy 100 ± 15%). The blood stability for 2.45 h at room temperature was found to be acceptable for imatinib. The % stabilities at LQC and HQC concentration were 100.72 and 99.27%, respectively (Table 7).

The comparative freeze-thaw stability calculated at the end of third freeze and thaw cycle ranged from 97.45 to 101.41% for imatinib (Table 8). The bench top stability in matrix for 6.15 h ranged from 97.75 to 100.23% for imatinib (Table 8). The stocks solution of imatinib was found stable for 21 days when stored in refrigerator between 1 and 10°C. The percent stability of stock solutions of imatinib and imatinib-D4 were found out to be 101.63 and 99.15%, respectively. The processed samples were found to be stable in auto sampler at 10°C for 100.87 h. The plasma samples were found to be stable when stored below -15°C and below -50°C for a period of 183 days (Table 8). Long-term stability for imatinib ranged from 103.09 to 107.18% (Table 8). The working solutions of imatinib and IS were found stable for 16 h at room temperature.

### Result of pharmacokinetic studies

#### Bioavailability studies

Validated method was employed to determine the plasma concentration of imatinib in samples of crossover study, using single dose of GLEEVEC (imatinib mesylate) 400 mg tablets (containing 400 mg of imatinib [as imatinib mesylate]) of Novartis Pharmaceuticals Canada Inc., and test formulation (containing 400 mg of imatinib [as imatinib mesylate]) of Sun Pharmaceuticals was administered to Indian subjects. The mean pharmacokinetic parameters, C_max_, T_max_ and AUC0–24 for test and reference product were mentioned in Table 9. Based on regulatory recommendation [25], the study matrix selected was plasma as imatinib is around 95% bound to plasma proteins.

The mean C_max_ for GLEEVEC in 26 Indian subjects was 1917.543 ng/ml while mean C_max_ for test formulation was 2055.983 ng/ml. The intrasubject variability (expressed in terms of % CV) was found to be as low as 28.40% for GLEEVEC (reference) and 26.27% for test formulation. In addition, an exploratory study was done to find bioavailability of reference product 30 European subjects at the same dose as that given to Indian Subjects. The pharmacokinetic profile of imatinib (test and reference) in Indian volunteers is shown in Figure 5.

The mean C_max_ for reference drug was 1716 ng/ml in European subjects and the intrasubject variability was comparable with that of Indian subjects (25%). The mean C_max_ was found to be 10–15% lower in European subject as compared with Indian subject. Therefore, the racial effect on bioavailability of imatinib was deemed statistically insignificant. The minor difference in mean C_max_ of reference product among the Indian and European subjects might be due to the difference in BMI as the mean BMI of Indian subject was 21.26 ± 2.17 kg/m^2^ while that of European subjects was found to be 24.01 ± 2.52 kg/m^2^.

#### Incurred sample reanalysis

The incurred sample reanalysis was found to be 100% for imatinib (Table 10) and negligible difference from original analysis was noted for the first time as compared with reported methods.

#### Comparison to other methods

The method was developed in a way to separate N-desmethyl imatinib and N-oxide of imatinib from parent compound while most of the reported methods were limited to separation of N-desmethyl imatinib only [26,27]. Also, the method prevented phospholipid-based matrix effects on the analytes of interest. Matrix factor and Matrix Effect had been studied in different types of plasma (Normal plasma, Hemolyzed and Lipemic), thereby supporting the exploratory as well as the bioavailability studies. The results of Matrix Factor showed that ion suppression or enhancement from the plasma matrix components was nullified. Further, the validated method was used to study racial effect on bioavailability of imatinib in Indian and European volunteers for the first time. Finally, the stability studies had been carried out extensively.

## Conclusion

The uncertainty in disease management with new late-entrant (i.e., bioequivalent branded generics) has immense opportunities, provided safety and efficacy of drug gets proved through trials as this leads to cost–effective prescriptions get generated. Hence, a rapid, sensitive and selective LC-MS/MS method for the quantification of imatinib in human plasma was developed and validated according to the requirements laid down in international regulatory guidelines.

The assay was linear from 9.57 to 4,513.29 ng/ml. The sensitivity of this unique bioanlytical method was sufficient to characterize pharmacokinetic concentration profile till 72 h (>five half-lives). The selectivity and stability performance of the method improved as compared with reported assay techniques due to separation and adequate control of labile metabolites in biological matrix. Furthermore, extraction method employing innovative solid phase extraction achieved consistent high recoveries for imatinib and IS from human plasma, without compromising matrix interference/ion suppression though matrices range has been extended in current research.

The samples were treated with 1.25% orthophosphoric acid solution that led to breaking of drug-protein binding and improved recovery (>85%) during solid phase extraction. Extraction recovery was consistent with minimal interference from metabolites (or from matrix components). Imatinib was found to be stable in human plasma during sample preparation, analysis, including the sample storage phases.

The method was applied to pharmacokinetic study of single dose of GLEEVEC (imatinib mesylate) 400 mg tablets (containing 400 mg of imatinib [as imatinib mesylate]) of Novartis Pharmaceuticals Canada Inc., Canada, and Test formulation (containing 400 mg of imatinib [as imatinib mesylate]) of Sun Pharmaceuticals in Indian subjects. All volunteers were administered the drug at the same time point and a crossover study was undertaken to remove biasness. For matching the safety and efficacy with reference drug, bioavailability studies, as per recommendations for regulatory agencies, were performed. In sum, the clinical applications, not only be extended to an exploratory study (may be following a therapeutic drug monitoring approach) but also for the bioequivalence route of commercialization of generic formulation. Therefore, studies were conducted in fasting condition although labeling information recommends taking imatinib with food. In addition, an exploratory study was done to find bioavailability of Reference product in European subjects. The study was conducted in Indian and European Volunteers. The intrasubject variability of European subjects was comparable with that of Indian subjects. The mean C_max_ was found to 10–15% lower in European subjects as compared with Indian subjects due to the difference in BMI as the mean BMI of Indian subjects.

**Table 1.** **Demographic profile of volunteers completing the study (Indian and European).**

**Demographic profile**	**Indian volunteers (n = 24)**	**European volunteers (n = 30)**
Gender	Male (100%)	Male (100%)
Age (years)	27.58 ± 4.92	24.7± 3.80
Height (cm)	164.23 ± 6.09	176.10 ± 6.15
BMI (kg/m^2^)	21.26 ± 2.17	24.01 ± 2.52
Weight (kg)	57.30 ± 6.09	74.78 ± 11. 22

**Table 2.** **Optimized mass spectrometer parameters for imatinib using API–3200 mass spectrometer.**

**Source/gas parameter**	**Compound parameter (eV)**
Curtain gas: 40^†^	Declustering potential: 65
Collisionally activated dissociation (CAD) gas: 3^†^	Entrance potential: 6
Ion spray voltage: 5000 eV	Collision energy: 38
Ion source gas1: 50^†^	Collision cell exit potential: 5
Ion source gas2: 50^†^	Resolution (Q1/Q3): unit/unit
Temperature: 550°C	
Interface heater: on	

^†^Typical values based on settings and scales defined in software application to control gas parameters.

**Table 3.** **Selectivity test for imatinib and imatinib-D4 in different lots of plasma.**

**Sample name**	**Imatinib**		**Imatinib-D4**	
	**Area**	**Area (%)**	**Area**	**Area (%)**
Blank 1	412	2.409	409	0.023
Blank 2	220	1.287	242	0.014
Blank 3	178	1.041	211	0.012
Blank 4	80	0.468	263	0.015
Blank 5	218	1.275	181	0.01
Blank 6	110	0.643	227	0.013
Blank 7^†^	154	0.901	87	0.005
Blank 8^‡^	163	0.953	118	0.007
LOQ–1	17559	NAP	1823001	NAP
LOQ–2	17296	NAP	1815286	NAP
LOQ–3	16403	NAP	1782262	NAP
LOQ–4	17277	NAP	1778668	NAP
LOQ–5	17164	NAP	1769828	NAP
LOQ–6	16900	NAP	1733432	NAP
Mean	17099.8	–	1783746.2	–
SD(±)	402.57	–	32523.6	–
%CV	2.35	–	1.82	–
n	6	–	6	–

^†^Hemolyzed blank.

^‡^Lipemic blank.

CV: Coefficient of variation; NAP: Not applicable; SD: Standard deviation.

**Table 4.** **Intra- and inter-batch precision and accuracy of imatinib.**

**Quality control level**	**Nominal concentration (ng/ml)**	**Intraday (n = 12)**			**Interday (n = 18)**		
		**Mean (ng/ml)**	**CV (%)**	**Accuracy (%)**	**Mean (ng/ml)**	**CV (%)**	**Accuracy (%)**
LLOQC	9.58	9.846	3.14	102.77	9.704	3.60	101.30
LQC	23.41	22.883	1.42	97.75	22.716	1.81	97.04
MQC	1800.91	1829.983	1.86	101.61	1835.620	1.65	101.93
HQC	3601.82	3705.032	1.75	102.87	3693.256	1.78	102.54

CV: Coefficient of variation; HQC: High qulaity control; LLOQC: Quality control at limit of quantification; LQC: Low quality control; MQC: Medim quality contol.

**Table 5.** **Percentage recovery of imatinib and imatinib-D4 in human plasma.**

**QC**	**Imatinib**	**Imatinib-D4**
LQC	80.86%	
MQC	93.59%	
HQC	87.57%	
Mean recovery	87.338%	87.87%
SD(±)	6.3657	
%CV	7.29	
n	3	

CV: Coefficient of variation; HQC: High qulaity control; IS: Internal standard; LLOQC: Quality control at limit of quantification; LQC: Low quality control; MQC: Medim quality contol.

**Table 6.** **Matrix effect and matrix factor for imatinib in six different lots of human plasma.**

**Lots of human plasma**	**Matrix effect**		**Matrix factor**					
	**LOQQC 9.58 ng/ml^†^**	**HQC 3601.82 ng/ml^†^**	**LQC**		**MQC**		**HQC**	
	**Calculated concentratin (ng/ml)**	**Calculated concentratn (ng/ml)**	**Using peak area response**	**Using analyte/IS peak area ratio**	**Using peak area response**	**Using analyte/IS peak area ratio**	**Using peak area response**	**Using analyte/IS peak area ratio**
Matrix lot #1	9.53	3603.46	1.13	1.02	1.07	1.03	1.04	1.01
	9.10	3557.21						
Matrix lot #2	9.12	3534.31	1.10	1.01	1.07	1.03	1.02	1.01
	8.95	3624.25						
Matrix lot #3	10.70	3602.58	1.11	1.03	1.06	1.02	1.00	1.00
	9.11	3575.45						
Matrix lot #4	9.05	3605.68	1.10	1.02	1.07	1.04	0.98	1.00
	8.97	3481.01						
Matrix lot #5^‡^	9.18	3506.62	1.06	1.00	1.04	1.02	0.96	0.99
	9.19	3556.62						
Matrix lot #6^§^	9.15	3516.40	1.02	1.01	1.02	1.02	0.98	1.00
	8.91	3515.64						
Mean	9.247	3556.603	1.086	1.013	1.056	1.027	0.996	1.003
SD(±)	0.4845	46.3716	0.0400	0.0103	0.0189	0.0061	0.0306	0.0108
%CV	5.24	1.30	3.68	1.02	1.79	0.60	3.07	1.08
n	12	12	6	6	6	6	6	6
%Nominal	96.52	98.74						

^†^Nominal concentration (ng/ml).

^‡^Hemolyzed plasma lot.

^§^Lipemic plasma lot.

CV: Coefficient of variation; HQC: High qulaity control; IS: Internal standard; LLOQC: Quality control at limit of quantification; LQC: Low quality control; MQC: Medim quality contol; SD: Standard deviation.

**Table 7.** **Sample collection process stability of imatinib.**

**Number of samples (n=4)**	**LQC**		**HQC**	
	**Stability samples**	**Comparison samples**	**Stability samples**	**Comparison samples**
	**Area ratio (analyte/IS)**	**Area ratio (analyte/IS)**	**Area ratio (analyte/IS)**	**Area ratio (analyte/IS)**
1	0.0248	0.0242	3.9210	3.9821
2	0.0245	0.0243	3.9921	3.9769
3	0.0245	0.0243	3.8916	3.9990
4	0.0239	0.0242	4.0109	3.9735
Mean	0.02443	0.02425	3.95390	3.98288
SD(±)	0.000377	0.000058	0.05678	0.011317
%CV	1.55	0.24	1.44	0.28
n	4	4	4	4
%Stability	100.72		99.27	

CV: Coefficient of variation; HQC;High quality control; IS: Internal standard; LQC: Low quality control; SD: Standard deviation.

**Table 8.** **Different stability parameters of imatinib.**

**Stability parameters**	**Nominal concentration (ng/ml)**	**Stability QC samples**		**Comparison QC samples**	
Bench top stability in matrix (6.15 h)	Mean	22.480	3696.288	22.468	3787.665
	SD(±)	0.3498	113.9628	0.352	24.3493
	%CV	1.56	3.08	1.57	0.64
	%Nominal	96.03	102.62	95.81	104.98
	n	4	4	4	4
Bench top stability during extraction (9.60 h)	Mean	22.193	3564.485	22.903	3681.380
	SD(±)	0.2660	61.3054	0.8096	41.3953
	%CV	1.20	1.72	3.54	1.12
	%Nominal	94.80	98.96	97.67	102.04
	n	4	4	4	4
Freeze thaw stability (three freeze thaw cycles, below -15°C)	Mean	22.745	3695.175	22.468	3787.665
	SD(±)	0.3283	71.3907	0.352	24.3493
	%CV	1.44	1.93	1.57	0.64
	%Nominal	97.16	102.59	95.81	104.98
	n	4	4	4	4
Freeze thaw stability (three freeze thaw cycles, below -50°C)	Mean	22.715	3684.818	22.468	3787.665
	SD(±)	0.4222	42.8389	0.3520	24.3493
	%CV	1.86	1.16	1.57	0.64
	%Nominal	97.03	102.3	95.81	104.98
	n	4	4	4	4
Auto sampler stability (100.87 h)	Mean	23.023	3695.965	22.720	3508.210
	SD(±)	0.2301	87.256	0.2696	36.4595
	%CV	1	2.36	1.19	1.04
	%Nominal	98.34	102.61	96.89	97.24
	n	4	4	4	4
Long-term stability below -15°C (183 days)	Mean	23.998	3442.073	22.438	3336.62
	SD(±)	0.3456	56.969	0.0806	28.4049
	%CV	1.44	1.66	0.36	0.85
	%Nominal	102.51	95.56	95.64	92.43
	n	4	4	4	4
Long-term stability below -50°C (183 days)	Mean	23.253	3432.1	22.438	3336.62
	SD(±)	0.2916	33.309	0.0806	28.4049
	%CV	1.25	0.97	0.36	0.85
	%Nominal	99.33	95.29	95.64	92.43
	n	4	4	4	4
Stock solution stability (21 days, stock solutions were stored in refrigerator temperature 1–10°C)	%Stability for imatinib	101.63			
	%Stability for imatinib-D4	99.15			
Short-term stability (8.46 h at room temperature)	%Stability for imatinib	101.88			
	%Stability for imatinib-D4	100.96			

CV: Coefficient of variation; QC: Quality control; SD: Standard deviation..

**Table 9.** **Pharmacokinetic parameter of imatinib (test and reference) in Indian volunteers.**

**Parameters**	**Test**	**Reference**
Mean C_max_ (ng/ml)	2055.983 (%CV: 26.27)	1917.543 (%CV: 28.40)
AUC _0-∞_	35293.60776 (%CV: 25.62)	36521.09140 (%CV: 25.46)
Mean T_max_ (h)	3.0	4.50
n	24	24

CV: Coefficient of variation.

**Table 10.** **Confirmatory incurred sample reanalysis of imatinib.**

**Incurred sample reanalysis details**	**Imatinib**
Total number of samples taken for incurred sample reanalysis	120
Number of samples meeting the acceptance criteria (i.e., % difference between the original and reanalyzed value must be within 20%)	120
Percentage of samples meeting the acceptance criteria	100.00

**Box 1.**  **Inclusion & exclusion criteria for recruitment of subjects.**

**Inclusion criteria**

Be in the age range of 18–45 years
Have their height/weight ratio within 15% of the values given on a standard height/weight table (e.g., The Life Insurance Corporation of India height/weight chart)
Be in the BMI range of ≥18.5–<30.0 kg/m^2^
Have voluntarily given written informed consent to participate in this study
Have hemoglobin level male: >12.0 g/dl (deciliter), female: >11.0 g/dl
Be of normal health as determined by medical history and physical examination of the subjects performed within 28 days prior to the commencement of the study
If female and:
  Of childbearing potential, is practicing an acceptable method of birth control for the duration of the study as judged by the investigator(s), such as condoms, foams, jellies, diaphragm, intrauterine device, or abstinence; or
  Is postmenopausal for at least 1 year; or
  Is surgically sterile (bilateral tubal ligation, bilateral oophorectomy/hysterectomy)

**Exclusion criteria**

Subject has history of hypersensitivity to imatinib or to any other related drug
Subject has history of musculoskeletal pain including myalgia, arthralgia, bone pain
Subject has history of peripheral edema or GI bleeding
Subject has history of intake of steroids or any immunosuppressants in a month preceding the study
Subject has any evidence of organ dysfunction or any clinically significant deviation from the normal in physical or clinical determinations
Clinically abnormal electrocardiogram (ECG) or hematological and biochemical parameters that is/are outside acceptable limits and is/are judged clinically significant by investigator
Investigations with blood samples of the subject show presence of disease markers of HIV 1 or 2, hepatitis B or C viruses or syphilis infection
Investigations with urine samples of the subject shows clinically abnormal chemical and microscopic examination of urine defined as presence of red blood cell, white blood cell (>4/high power field), glucose (positive) or protein (positive)
Subject has history of serious medical illnesses including but not limited to gastrointestinal, hepatic, renal, cardiovascular, pulmonary, neurological or haematological disease, diabetes, glaucoma, any serious, potentially life-threatening illness
Inability to communicate well (i.e. language problem, poor mental development, psychiatric illness or poor cerebral function) that may impair the ability to provide, written informed consent
Subject is a regular smoker, who smokes more than ten cigarettes daily or has difficulty abstaining from smoking for the duration of each study period
Subject has history of drug dependence or excessive alcohol intake on a habitual basis or has difficulty in abstaining or found positive in alcohol breath test before admission in period
Use of any medication within 30 days prior to admission of this study
Subject has participated in a clinical trial within 3 months (90 days) preceding admission of this study (except for the subjects who dropout/withdrawn from the previous study prior to period I dosing)
Consumption of alcohol for 48 h prior to admission
Consumption of grapefruit juice and or grape fruit supplements containing products for 48 h prior to admission
Subject has problem(s) in complying with the study protocol
Female volunteers demonstrating a positive pregnancy test
Female volunteers who are currently breast-feeding
Subject has donated and/or lost more than 350 ml of blood in the past 3 months (90 days) prior to admission

**Executive summary**

A novel, accurate and high-throughput tandem mass spectroscopic method has been developed and validated for determination of imatinib, used in treatment of chronic myeloid leukemia.
Imatinib was estimated meeting the challenges of interferences from endogenous matrices as well as separation from labile metabolites had been achieved.
The results of matrix factor showed that ion suppression or enhancement from the plasma matrix components was nullified.
Stability studies had been carried out extensively and results were deemed acceptable.
The intrasubject variability in pharmacokinetic profile for European subjects was comparable with that of Indian subjects.
